# Predictive value of Neutrophil-to-Lymphocyte Ratio and Platelet-to-Lymphocyte Ratio in Patients with Vaginitis

**DOI:** 10.12669/pjms.37.1.2774

**Published:** 2021

**Authors:** Eren Pek, Fatma Beyazit, Nilay Sen Korkmaz

**Affiliations:** 1 Dr. Eren Pek, Assistant Professor, Department of Obstetrics and Gynecology, Faculty of Medicine, Canakkale Onsekiz Mart University, Turkey; 2 Dr. Fatma Beyazit, Associate Professor, Department of Obstetrics and Gynecology, Faculty of Medicine, Canakkale Onsekiz Mart University, Turkey; 3 Dr. Nilay Sen Korkmaz, Assistant Professor, Department of Medical Pathology, Faculty of Medicine, Afyonkarahisar Health Sciences University, Turkey

**Keywords:** Bacterial vaginosis, Vulvovaginal candidiasis, Neutrophil-lymphocyte ratio, Platelet-lymphocyte ratio, Mean platelet volume

## Abstract

**Objectives:**

This study was conducted to evaluate the diagnostic value of platelet-to-lymphocyte ratio (PLR) and neutrophil-to-lymphocyte ratio (NLR) in vaginitis patients.

**Methods:**

This cross-sectional retrospective study was performed in Afyon Dinar State Hospital between July 2016 to August 2017. A total of 64 bacterial vaginosis (BV) patients, 66 vulvovaginal candidiasis (VVC) patients and 65 age-matched control subjects were enrolled. NLR, PLR, mean platelet volume (MPV), red cell distribution width (RDW) and other conventional inflammatory marker values were recorded for all patients before and after treatment.

**Results:**

In the BV group, NLR values were found to be elevated compared to VVC and healthy controls [2.9 (1.2-14.7), 2.1 (1.1-11.7) and 2.1 (0.8-7.0), respectively] (p=0.008). Although not found to be statistically significant, the median NLR levels of BV patients decreased from 2.9 (1.2-14.7) to 2.4 (1.2-7.0) after treatment. PLR levels did not show a statistically significant difference between the three groups (p=0.970). The cut-off value of the NLR for BV was 2.19, with 67.2% sensitivity and 63.8% specificity.

**Conclusions:**

The present study demonstrated that NLR levels are elevated in bacterial vaginosis and NLR levels can be used as a reflection of systemic inflammatory response in vaginosis patients.

## INTRODUCTION

Vaginitis either bacterial or fungal origin is one of the commonest reproductive tract infections amongst sexually active women and characterized with inflammation of the vagina as a result of infectious agents. It usually presents with vaginal discharge, vulvar itching, irritation, odour and dysuria. The most common etiologic agents of infectious vaginitis are bacterial vaginosis (BV), candidiasis and trichomoniasis, which generally account for 90% of all etiologies.^1^ Although clinical differentiation of various forms of infectious vaginitis is unreliable, there are certain individual symptoms and signs that are associated with the disease. Unfortunately, usual symptoms of infectious vaginitis are nonspecific and diagnosis without laboratory confirmation can lead to inappropriate medication.^2^ Therefore, adjunctive use of additional diagnostic methods that will be more sensitive and specific for the determination of disease presence and its extent is needed, preferably in daily gynaecologic practice.

Sequential hematologic system alterations following induction of systemic inflammatory response has been found to be an independent marker of disease diagnosis and prognosis in several types of inflammatory disorders. In this context, neutrophil-lymphocyte ratio (NLR) and platelet–lymphocyte ratio (PLR) is coming into use as a marker of systemic inflammation in studies on several inflammatory disease conditions including ulcerative colitis, tuba-ovarian abscess, acute appendicitis, subacute thyroiditis, chronic hepatitis B and C infection and sepsis.^3^-^7^ Moreover, NLR acts as a balance between the active inflammatory component and the regulatory/protective component in distinct disease conditions and has been found as a valuable index for predicting clinical outcomes in inflammatory and neoplastic diseases such as Alzheimer’s disease, acute appendicitis and ulcerative colitis.^3^,^5^,^8^

Some other hematologic indices such as mean platelet volume (MPV), red blood cell distribution width (RDW), and platelet distribution width (PDW) have also been studied as novel markers of inflammation in several infectious diseases.^9^-^11^ Unfortunately, knowledge regarding the association between hematologic parameters and the presence of infectious vaginitis either bacterial or fungal origin is scarce. Recognizing the need for detection and etiologic discrimination of different infectious vaginitis types, this study was performed in order to determine whether these simple hematologic markers are altered in patients with infectious vaginitis and if these parameters are affected by anti-infective treatment.

## METHODS

**Fig.1:**
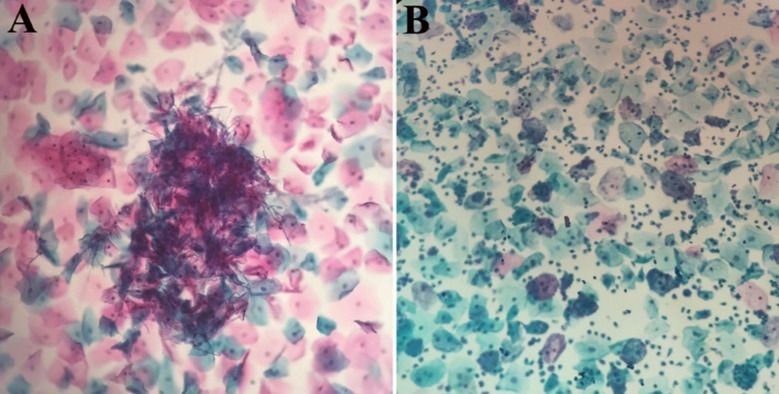
Microscopic examination of smear specimens showing A) pseudohyphae and yeast forms of candida B) squamous cells covered with cocobacilli, resulting in clue cells (papanicolauu staining; x200).

A retrospective review of the available medical records of vaginosis patients with bacterial or fungal origin (proven either clinically or microscopically), admitted to Afyon Dinar State Hospital from July 2016 to August 2017 and who had received anti-infective treatment, and were included in the present study. Diagnosis and the differentiation of BV from vulvovaginal candidiasis (VVC) was based primarily on clinical examination with the support of microscopic findings. Although both types of the vaginitis have similar clinical manifestations including edema and erythema of the vulva and the vagina, in VVC vaginal discharge typically resembles cottage cheese. Based on the conventional method, smear samples from cervix were collected and prepared either with a brush or spatula by a gynaecologist and fixed on glass slides. Afterwards, smears were evaluated microscopically by an independent specialist. The diagnosis of BV was based on the identification of coccobacilli or clue cells on microscopy, and the diagnosis of VVC was done if fungal hyphae or budding yeasts were present in wet mount (Fig.1). The study was approved by the local ethics committee of Canakkale Onsekiz Mart University (Approval No: 2011-KAEK-27/2017-E.70415) and was conducted in accordance with the guidelines of the Helsinki Declaration.

The following data was extracted from hospital medical records before and after treatment for each patient: age, medical history, presence or absence of symptoms, laboratory tests and eventual diagnosis. Complete blood cell count parameters [White blood cell (WBC), neutrophil, lymphocyte, hemoglobin, hematocrit, platelet, MPV and RDW], C-reactive protein (CRP), and erythrocyte sedimentation rate (ESR) of the patients were obtained from the patients’ files and recorded in an information Form developed for this purpose. The NLR was calculated by dividing the absolute neutrophil count by the absolute lymphocyte count; PLR was calculated by dividing the platelet count by the lymphocyte number. All CBC analysis was performed in a hematology laboratory of the same hospital with a Horiba ABX Pentra DF 120 automated analyser.

Women were excluded from the study if they had a history of receiving anti-infective treatment or local vaginal antimicrobial treatment within the preceding 10 days. Moreover, women with a positive pregnancy test, systemic diseases including hematologic disorders, acute or chronic inflammatory disorders, NSAID or oral contraceptive consumption before hospital admission, were also excluded.

### Statistical Analysis

The Statistical Package for Social Sciences (SPSS) 20.0 for Windows was used to analyse the data. Continuous variables were tested for normality by the Kolmogorov-Smirnov test. Values were presented as mean ± standard deviations. All normally distributed data were analysed using one-way ANOVA for continuous variables. Data found to be non-normally distributed were analysed using Kruskal–Wallis and Mann–Whitney (with Bonferroni correction) statistical tests. Receiver operating characteristic (ROC) curve analysis was used to identify optimal cut-off values of NLR and PLR to identify with maximum sensitivity and specificity for the detection of BV. P values below 0.05 were considered as statistically significant. Two-tailed Spearman correlation analysis was performed to determine the correlation coefficient (r) for comparisons between NLR and other variables.

## RESULTS

The study consisted of 64 patients with BV, 66 patients with VVC, and 65 healthy controls with a mean age of 33.3±9.9, 32.7±10.3 and 31.2±10.2 (Table-I). There were no statistically significant differences between the ages of the study participants (p=0.443).

**Table-I:** Demographic characteristics and laboratory values of the patients and controls.

	Bacterial vaginosis (n=64)	Vulvovaginal candidiazis (n=66)	Healthy controls (n=65)	p
Age	33.3±9.9	32.7±10.3	31.2±10.2	0.443
Hemoglobin (g/dl)	11.7±1.5	12.4±1.3	12.2±1.7	0.052
WBC (/mm^3^x10^3^)	8.6±2.4	8.2±3.2	8.0±2.5	0.128
Platelet (/mm^3^x10^3^)	262.6±62.3	279.3±58.6	282.5±71.8	0.054
MPV (fL)	8.9 (7.3-12.9)	9.1 (6.7-10.6)	9.0 (6.9-11.3)	0.973
PDW	16.3 (11.8-27.8)	15.9 (9.8-19.8)	17.0 (10.8-25.3)	0.874
RDW (%)	15.6 (11.7-24.5)	15.2 (12.4-24.5)	15.2 (12.3-26.2)	0.231
NLR	2.9 (1.2-14.7)	2.1 (1.1-11.7)	2.1 (0.8-7.0)	0.008*
PLR	128.0 (47.9-352.3)	128.9 (60.9-376.0)	126.6 (46.4-400.0)	0.970
CRP (mg/L)	0.3±0.5	0.3±0.5	0.7±2.0	0.345
ESR (mm/h)	10.6±4.4	10.9±3.2	10.6±3.9	0.666

* Bacterial vaginosis vs vulvovaginal candidiazis (p=0.014) and controls (p=0.004).

The mean WBC values were found to be 8.6±2.4 x103/µl for BV, 8.2±3.2 x103/µl for VVC, and 8.0±2.5 x103/µl for controls (p=0.128). No statistically significant differences in hemoglobin, platelet, MPV, PDW, RDW, and PLR were observed between groups (p>0.05) (Table-I). The median NLR values of BV, VVC and controls were 2.9 (1.2-14.7), 2.1 (1.1-11.7) and 2.1 (0.8-7.0) respectively (p= 0.008). The NLR values of BV patients were significantly higher from VVC (p=0.014) and controls (p=0.004). No significant difference was observed between VVC and controls in respect to NLR levels (p>0.05). When the PLR values of the three groups were compared, no statistically significant difference was observed between the groups (p=0.970). The demographics and descriptive statistics for all variables in the study are presented in Table-I.

**Fig.2:**
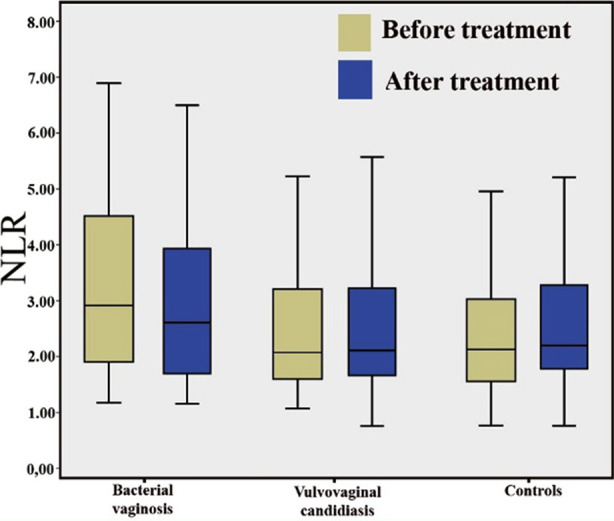
Box plot presentation of the patients and controls before and after treatment according to neutrophil-lymphocyte ratio (NLR) values.

When the hematologic and inflammatory parameters of BV and VVC patients were analysed before and after treatment separately, the median NLR levels of BV patients were found to have decreased from 2.9 (1.2-14.7) to 2.4 (1.2-7.0). Although this decrease was not found to be significant (p=0.929), no such trend was observed in respect to NLR values in VVC patients (Fig.2). The PLR levels also did not differ before and after treatment in patients with BV (p=0.547) and VVC (p=0.438) patients. Only CRP and ESR levels were significantly different after treatment (Table-II).

**Table-II:** Comparison of NLR, PLR, MPV and RDW with other inflammation markers at admission and after treatment.

	Bacterial vaginosis (n= 64)			Vulvovaginal candidiazis (n= 66)		

At admission	After treatment	p	At admission	After treatment	p

NLR	2.9 (1.2-14.7)	2.4 (1.2-7.0)	0.929	2.1 (1.1-11.7)	2.1 (0.8-5.8)	0.425
PLR	128.0 (47.9-352.3)	127.2 (54.9-356.2)	0.547	128.9 (60.9-376.0)	134.7 (74.1-226.1)	0.438
MPV (fL)	8.9 (7.3-12.9)	9.1 (7.2-12.3)	0.435	9.1 (6.7-10.6)	9.0 (7.4-10.9)	0.655
RDW (%)	15.6 (11.7-24.5)	15.9 (11.6-21.0)	0.740	15.2 (12.4-24.5)	15.1 (12.2-20.0)	0.646
CRP (mg/L)	0.3±0.5	0.2±0.4	<0.001	0.3±0.5	0.1±0.3	0.010
ESR (mm/h)	10.6±4.4	8.8±2.6	<0.001	10.9±3.2	9.3±2.0	<0.001

Following analysis of the distribution of NLR values, ROC curves were constructed to assess the ability of NLR to differentiate BV from VVC (Table-III). The optimum NLR cut-off point for BV was 2.19 with a sensitivity, specificity, positive predictive value (PPV) and negative predictive value (NPV) of 67.2%, 63.8%, 59.3% and 62.1%, respectively (AUC: 0.648). The same analysis for PLR is summarized in Table-III.

**Table-III:** Overall accuracy and ROC analysis of NLR and PLR to differentiate bacterial vaginosis from vulvovaginal candidiasis.

	Sensitivity (%)	Specificity (%)	PPV (%)	NPV (%)	AUC	OA

NLR (Cut off: 2.19)	67.2	63.8	59.3	62.1	0.648	60.5
PLR (Cut off: 119.34)	60.9	50.0	50.4	50.6	0.512	50.5

NPV: Negative predictive value; PPV: Positive predictive value; AUC: Area under curve; OA: Overall accuracy.

Spearman correlation analysis indicated that NLR values before treatment had a significant correlation with PLR (r=0.366, p<0.001). No significant correlation was observed between NLR and other hematologic or inflammatory markers.

## DISCUSSION

The elevated NLR levels demonstrated in this study could be the result of an activated immune response to bacterial infection which results in amplification of neutrophils and a decrease in lymphocyte counts. In inflammatory conditions several chemotactic molecules play important roles in the recruitment of neutrophils from blood into inflamed tissue and start a destructive tissue cascade.^3^ Bacterial infection is one of the main triggers that rapidly migrates the circulating neutrophils to the infected site, and the bone marrow increases neutrophil production to compensate neutrophil consumption.^12^ On the other hand, lymphocyte count usually decreases in inflammatory conditions caused by bacterial infections and in acute stress settings, secondary to increased levels of corticosteroids.^13^ For this reason, it is not surprising to detect NLR alterations in inflammatory disorders because of the presence of left shift indicating an increase of neutrophil consumption. However, no study in the literature has studied NLR and other hematologic indices in patients with vaginal infections. In a study by Seçkin et al.^14^ it was shown that NLR levels were elevated in patients with pelvic inflammatory disease (PID). Moreover, NLR was found to have the highest sensitivity and specificity in diagnosing PID with a similar diagnostic sensitivity and specificity as CRP. In the present study, we also identified important information both about the diagnostic yield and about the role of treatment on the inflammation using NLR alterations. Being a simple and inexpensive index of systemic inflammatory burden, we believe that a standardized cut-off value for NLR can be used for discriminating bacterial vaginosis from other infectious vaginitis etiologies, and can give a significant clue to the clinician for estimating treatment results in individual patients.

PLR has also been proposed as another indicator of systemic inflammation, although less frequently investigated than NLR in different studies. This is due to the growing evidence regarding the role of platelets as initial actors in the processes of inflammation and tissue repair. Platelets closely collaborate with all types of leukocytes. Chemotactic substances secreted from activated platelets accelerate the binding of leukocytes to the endothelial surface and their subsequent extravasation, and they may affect the inflammatory responses of leukocytes in both stimulating and inhibiting ways^15^ Chronic low-grade inflammation may cause an elevation in PLR, and increased PLR might be a sign of ongoing inflammation, which would eventually be associated with an increased risk of various conditions including coronary artery disease, solid organ tumours, and autoimmune disorders.^16^-^18^ Therefore, we hypothesized that PLR levels might be affected from systemic inflammation in vaginitis. However, we did not observe any alterations in PLR levels in both bacterial vaginosis and vulvovaginal candidiasis patients before and after treatment. This might be due to several reasons including sample size of the study population and the low-grade inflammation found in vaginitis patients.

In this study we also evaluated the role of two distinct inflammation-related hematologic indices, such as MPV and RDW in vaginosis patients. MPV is a simple marker showing platelet function and activation that can be determined by full blood count analysers as a part of the routine CBC test cycle which is commonly overlooked by the clinicians.^19^ It is influenced from inflammatory conditions and alterations have been reported in several disease conditions. RDW can also be measured from a standard full blood count and is a quantitative measure of anisocytosis, the variability in size of the circulating erythrocytes. Elevated RDW typically shows ineffective red cell production (such as iron deficiency, B12 or folate deficiency), hemolysis and after blood transfusion. Systemic inflammation might contribute to RDW elevation by not only impairing iron metabolism but also by inhibiting the production of, or response to, erythropoietin, or by shortening RBC survival.^20^ Both of these scores have been applied in many other types of inflammatory and neoplastic disorders including acute pancreatitis, allergic airway diseases, familial Mediterranean fever and cervical cancer.^21^,^22^ But to the best of our knowledge little is known about the impact of vaginal infection and anti-infective treatment on MPV and RDW scores. Therefore, the present data is the first demonstration that, apart from NLR, all other hematologic markers including MPV and RDW do not effect from chronic inflammation triggered by infectious agents causing vaginitis.

### Limitations of the study

The present study has some limitations, and their recognition should help refine future research efforts. First, the data has been collected from only one centre, which gives concerns about generalizing the results of this study. Second, due the retrospective nature of the study the data were not acquired in a standardized way, which could be regarded as a possible source of biases. And third, it is unclear from our study whether NLR and systemic inflammation have a cause and effect relationship.

## CONCLUSIONS

The present study for the first time revealed that patients with bacterial vaginosis have elevated NLR levels in comparison with vulvovaginal candidiasis and healthy controls. Evaluating NLR as an adjunctive marker for the future evaluation of bacterial vaginosis could aid in the diagnosis and monitoring progress in treatment. We therefore suggest that NLR, as a simple and inexpensive test, is a valuable tool for a rapid, at a glance assessment of BV.

### Authors’s Contribution:

**EP:** Designed, conceived, statistical analyses, data collection, is responsible and accountable for the accuracy or integrity of the work.

**FB:** Did manuscript writing, editing of manuscript.

**NSK:** Carried out the lab work.
